# Stable phase-shift despite quasi-rhythmic movements: a CPG-driven dynamic model of active tactile exploration in an insect

**DOI:** 10.3389/fncom.2015.00107

**Published:** 2015-08-21

**Authors:** Nalin Harischandra, André F. Krause, Volker Dürr

**Affiliations:** ^1^Department of Biological Cybernetics, Faculty of Biology, Bielefeld UniversityBielefeld, Germany; ^2^Cognitive Interaction Technology Center of Excellence (CITEC), Bielefeld UniversityBielefeld, Germany

**Keywords:** active tactile sensing, tactile exploration, central pattern generators, dynamic modeling, hair fields, insect antennae, non-linear oscillators, quasi-rhythmic oscillations

## Abstract

An essential component of autonomous and flexible behavior in animals is active exploration of the environment, allowing for perception-guided planning and control of actions. An important sensory system involved is active touch. Here, we introduce a general modeling framework of Central Pattern Generators (CPGs) for movement generation in active tactile exploration behavior. The CPG consists of two network levels: (i) phase-coupled Hopf oscillators for rhythm generation, and (ii) pattern formation networks for capturing the frequency and phase characteristics of individual joint oscillations. The model captured the natural, quasi-rhythmic joint kinematics as observed in coordinated antennal movements of walking stick insects. Moreover, it successfully produced tactile exploration behavior on a three-dimensional skeletal model of the insect antennal system with physically realistic parameters. The effect of proprioceptor ablations could be simulated by changing the amplitude and offset parameters of the joint oscillators, only. As in the animal, the movement of both antennal joints was coupled with a stable phase difference, despite the quasi-rhythmicity of the joint angle time courses. We found that the *phase-lead* of the distal scape-pedicel (SP) joint relative to the proximal head-scape (HS) joint was essential for producing the natural tactile exploration behavior and, thus, for tactile efficiency. For realistic movement patterns, the phase-lead could vary within a limited range of 10–30° only. Tests with artificial movement patterns strongly suggest that this phase sensitivity is not a matter of the frequency composition of the natural movement pattern. Based on our modeling results, we propose that a constant phase difference is coded into the CPG of the antennal motor system and that proprioceptors are acting locally to regulate the joint movement amplitude.

## 1. Introduction

A basic element of autonomous intelligent behaviors is the ability to actively explore the surrounding environment with different sensing systems (Beer, 2000; Nelson and MacIver, 2006; Prescott et al., 2011). In nature, such explorative behaviors are mostly related to locomotion, thus introducing a link between the actively moving sensory systems and the locomotive organs (e.g., Dürr et al., 2003; Egelhaaf et al., 2012). This is particularly the case in tactile sensing systems, where the receptive field of the sensor is limited to the width of a patch of hairs (as in the vertebrate whisker system) or to a single feeler (as in insect or crustacean antenna). Thus, efficient tactile exploration of the near-range environment requires continuous, rhythmic and adaptive active movements of the tactile sensor.

The main objective of this study is to devise a framework for the systematic study of adaptive changes in coordination of rhythmic sensor movements in behavior. For this purpose, we choose the active tactile system of walking insects as a paragon. Indeed, many animals use actively moveable tactile sensors to explore the near-range environment. While mammals like cats and rats use active whisker movements to detect and scan objects in the vicinity of their body (Cramer et al., 2007; Prescott et al., 2011), many walking insects and crustaceans continuously search and sample the space ahead with their antennae or feelers (Staudacher et al., 2005). In their ground plan, all higher insects carry two antennae with two actuated joints per antenna. The coordination of their rhythmic movements is important for at least three aspects of the active touch system: (i) for coordinated action of the two joints per antenna (inter-joint coupling), (ii) for coordinated search and sampling by the two antennae (inter-limb coupling), and (iii) for coordination of the sensors with movements of the head and legs (inter-segmental coupling). As such, the study and modeling of adaptive changes in coordination of insect antennal movements is of general interest to adaptive coordination of limbs, and of particular relevance to active sensing.

The Indian stick insect, *Carausius morosus* strongly relies on its antennae for near-range searching and sampling of encountered objects (Dürr, 2014). Stick insects show rhythmic tactile exploration behavior during locomotion, involving coordinated movements of two joints, the head-scape and scape-pedicel joint (Dürr et al., 2001; Krause et al., 2013). Moreover, they alter the coupling of the two joints per antenna either unilaterally or bilaterally, depending on walking direction (Dürr and Ebeling, 2005) and on whether touch events occur on one (Schütz and Dürr, 2011) or both antennae (Krause and Dürr, 2012). Upon antennal contact with an object, both the frequency and the amplitude of the rhythmic antennal movement are modulated, affecting both antennal joints in a context-dependent manner (Schütz and Dürr, 2011; Krause and Dürr, 2012). Finally, ablation of joint proprioceptors mainly affects the working-ranges of antennal joints but has no effect on the overall pattern of inter-joint coordination (Krause et al., 2013). To date, the neuronal network that generates rhythmic antennal movement has not been identified, but it has been localized in the brain and shown to be sensitive to the muscarinic acetylcholine agonist pilocarpine (Okada et al., 2009; Krause et al., 2013). Because of similarities in pharmacology, and of serial homology between legs and antennae, it is reasonable to assume that rhythmic movements of the antennae are driven by a set of coupled neural oscillators with one oscillator per joint, similar to what has been proposed for single leg control (Ekeberg et al., 2004).

Accordingly, the main objective of the present paper is to apply the concept of mutually coupled oscillators in a Central Pattern Generator (CPG) framework for modeling the quasi-rhythmical tactile exploration movements of insect antennae. In particular, we wanted to understand whether and how the experimentally observed effects of proprioceptor ablations could be emulated by a simple change of the controller parameters. Furthermore, we investigate the constraints of a stable phase difference in the presence of a variable, a quasi-rhythmic movement pattern with multiple frequency components. CPGs are biological neural networks that produce rhythmic outputs without rhythmical inputs (Grillner and Zangger, 1975; Marder and Bucher, 2001; Selverston, 2010). However, the CPG activity is constantly modulated by peripheral sensory feedback (both exteroceptive and proprioceptive) and activation from other regions of the nervous system, for instance, descending signals from the brain (Pearson, 1972; Büschges et al., 2008; Mulloney and Smarandache, 2010). Depending on the phenomena under study, CPG models have been designed at different levels of abstraction ranging from detailed biophysical models, to connectionist models, to abstract systems of coupled oscillators (for review, see Ijspeert, 2008). Given the sparse knowledge about the underlying physiological networks in the insect antennal system, here we use a system of coupled non-linear phase oscillators as the core of the controller model. The CPG model consists of two layers that can be considered as abstractions of a rhythm-generating- and pattern-forming network (Rybak et al., 2006). In addition to the computational model of the controller, we developed a 3D forward dynamic model of the active tactile system of the stick insect head and antennae. In our approach, we compare and evaluate a set of pattern-forming networks with different frequency characteristics. We can show that the model can capture the effect of proprioceptor ablations by changing the oscillator amplitudes only. Finally, we show the significance of the experimentally observed phase difference between the two antennal joints for tactile searching movements.

The computational framework and the model simulator will be useful for both neuroscientists and engineers interested in the design and control of tactile sensing systems. For neuroscientists, knowledge of the CPG configuration and the changes in the kinematics with respect to CPG parameters will give a means of identifying control requirements. This will shed new light on the available neurophysiological data regarding neural control of antennal movements, inter-joint coordination in particular. For roboticists, the identified control system or algorithm can be used for designing efficient and adaptive controllers for bio-inspired and biomimetic active tactile systems (Lewinger et al., 2005; Prescott et al., 2009; Pearson et al., 2011; Patanè et al., 2012; Sullivan et al., 2012; Harischandra and Dürr, 2013; Hoinville et al., 2014).

## 2. Materials and methods

### 2.1. Experimental data

Kinematic data has been taken from the motion capture experiments carried out on adult female stick insects of the species *Carausius morosus* (Krause et al., 2013). Among other things, that study showed that antennal inter-joint coordination is persistent, both in intact, unperturbed spontaneous movements during walking and in pharmacologically induced movements (though with lower cycle frequency). In both situations, there was a characteristic phase lag of the proximal head-scape (HS) joint with respect to the distal scape-pedicel (SP) joint. Here, we use the antennal joint angle time courses measured by Krause et al. (2013) for rhythmic searching movements of unrestrained, straight walking stick insects. From these time courses, we obtained the frequency spectra in order to identify plausible pattern generating descriptors (see Section 2.3).

Another important finding of recent physiological experiments concerns the proprioceptive control of antennal movement pattern, in particular the role of antennal hair fields. Hair fields are rows or patches of mechanoreceptive hairs located next to the joint membranes of insect limbs or antennae. They encode relative movement of the two adjoining segments (Okada and Toh, 2001; Krause et al., 2013). In stick insects, it has been shown that the ablation of antennal hair fields (proprioceptors) had no effect on the overall pattern of inter-joint coordination but could affect the magnitude of the phase-lag. However, ablation of dorsal hair fields affected the working-ranges of both antennal joints. A considerable expansion of the dorsal HS working range could be seen after ablating the dorsal scapal hair plate (Krause et al., 2013).

### 2.2. Skeletal model and forward dynamics simulation

**Figure 1:**
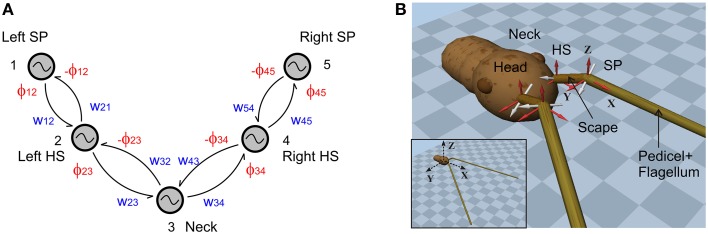
**CPG configuration and the 3D skeletal model**. **(A)** The CPG network consists of two oscillators per antenna and a single neck oscillator. Coupling strengths and phase differences are denoted by *w*_*ij*_ and ϕ_*ij*_, respectively (see **Table 1**). HS and SP stand for head-scape and scape-pedicel. **(B)** Two snap shots of the simulator show the skeletal model of the stick insect head and antennal system. Note the non-orthogonal, slanted joint axes (white) for each antennal joint. Scape and pedicel+flagellum are the proximal and the distal segments of each antenna, respectively. The external coordinate system is shown in the inset. The local coordinate systems for each antennal joint are shown in red, with their origins being located at the corresponding joint position.

The skeletal model underlying the forward dynamics simulation comprises the prothorax, the head and the two antennae. Each modeled antenna is composed of two segments: the *scape* and the *pedicel-flagellum*. Due to the passive nature of the joint between the pedicel and the flagellum, they are lumped together as a single segment in the model. The segments are connected via hinge joints, each one with a slanted axis of rotation and one degree of freedom (DOF, see Figure 1B). The head is connected to the prothorax via a neck joint with a vertical hinge joint axis. As a result, the single DOF of the neck allows for yaw rotations in the horizontal plane, only. Real stick insects have at least one other DOF in the neck, that is pitch. Though detailed motion analysis of the neck joints have not been carried out yet, Theunissen et al. (2015) found that stick insects adjust head pitch during climbing, so as to contribute to head stabilization. Since head pitch does not appear to be very rhythmical, we neglected it in the present version of the skeletal model, in order to simplify the control scheme.

While all antennal segments were modeled as rigid, uniform cylinders, the head was spherical. Geometrical (kinematic) parameters of the model were chosen such that they match the average values given by Dürr et al. (2001) and Krause and Dürr (2004). However, to overcome the numerical instabilities when simulating very small inertial components, the mass of each segment was set to a higher value (about factor of 10) compared to the real values. Additionally, the geometrical parameters were also scaled by a factor of 10. Table 1 shows all relevant physical parameters of the skeletal model. Position coordinates and axes of rotation for the HS and SP joints are given for the left antenna only, assuming bilateral symmetry with respect to the sagittal plane (*y* = 0) for the corresponding values of the right antenna. Note that, because the axes are specified in the external coordinate system, the position data are valid only for the initial configuration where both antennae are outstretched in the horizontal plane at an angle of 10° facing medially. The center of the spherical head, in its initial position, is located at the origin of the external coordinate system (inset of Figure 1B). The rotational axes for the joints are given in local coordinate systems as shown in Figure 1B. Note that the two antennal joint axes are slanted with regard to the sagittal plane and non-orthogonal to each other (Mujagic et al., 2007). This is different from closely related insect orders which have orthogonal joint axes that are aligned with the horizontal and sagittal planes of the body (Staudacher et al., 2005).

**Table 1:** **Parameters of the skeletal model**.

**Joint**	**Head-scape**	**Scape-pedicel**	**Neck**
Position	(0.0174, −0.0083, 0.0100)	(0.0342, −0.0066, 0.0100)	(−0.015, 0.0, 0.0)
Axis	(−0.1480, 0.8430, 0.5140)	(−0.1530, 0.8690, −0.4690)	(0.0, 0.0, 1.0)
**Segment**	**Scape**	**Pedicel-flagellum**	**Head**
Length (mm)	17.0	263.0	40.0 (diameter)
Mass (mg)	3.0	12.5	200.0

In this study, all joints were abstracted to be velocity-controlled servo-motors. The model was tested in a computer-simulated environment where we used a network of non-linear oscillators (see next section) to drive the motors, which in turn generated the movements of the skeletal model. While this abstraction neglects any effects caused by muscle contraction dynamics, it is suitable for analyzing the effects arising through the coupling of multiple CPGs, which is the main focus of our study.

### 2.3. Central pattern generator

The central pattern-generating network used for the control of the skeletal model has two levels (networks) of components per joint oscillator. The first level sets the timing (or phase) and amplitude information of the reference position of each antennal joint and the neck. Together, these components are the *Rhythm Generating* network, abstracting a network of neural oscillators. It is composed of *Hopf oscillators*, connected by means of diffusive couplings. The state variables of the individual oscillators are given by the following equations in phase space.

(1) $$\begin{array}{rcl} {ṙ}_{i} & = & \gamma_{i}\left(\mu_{i}^{2}-r_{i}^{2}\right)r_{i} \end{array}$$

(2) $$\begin{array}{rcl} {\overset{{}^{\circ}}{\theta }}_{i} & = & 2\pi v_{i}+\underset{j}{\sum }w_{ij}\sin\left(\theta_{j}-\theta_{i}-\phi_{ij}\right) \end{array}$$

Here, *r*_*i*_ and θ_*i*_ represent the amplitude and phase of the *i*th oscillator, respectively. *v*_*i*_ and μ_*i*_ determine the intrinsic frequency and amplitude, and γ_*i*_ is a positive constant which determines the speed of convergence to the desired amplitude. The weights between oscillators and the phase bias were set by *w*_*ij*_ and ϕ_*ij*_, respectively. Note that in this model, the phase-response curve of the coupling term appears explicitly in Equation (2), as the sine of the phase difference (θ_*j*_ − θ_*i*_) plus an offset of −ϕ_*ij*_. Thus, (*r*_*i*_, θ_*i*_) describe the *Rhythm Generating* network of the CPG.

To include the desired offset *C*_*i*_ in the oscillator, an additional state variable is introduced to the dynamical system:

(3) $$\begin{array}{l} {ċ}_{i}=\gamma_{i}^{c}\left(C_{i}-c_{i}\right) \end{array}$$

where, $\gamma_{i}^{c}$ is a positive constant determining the convergence speed of *c*_*i*_ to the desired offset, *C*_*i*_. Hence the driving signal, *x*_*i*_, of the *i*th oscillator can be defined as:

(4) $$\begin{array}{l} x_{i}=c_{i}+r_{i}F_{i}\left(\theta_{i},f_{k}\right) \end{array}$$

where

(5) $$\begin{array}{l} F_{i}\left(\theta_{i},f_{k}\right)=M_{i}\overset{L}{\underset{n=0}{\sum }}\left[a_{n}\cos\left(n\theta_{i}\;f_{k}\right)+b_{n}\sin\left(n\theta_{i}\;f_{k}\right)\right] \end{array}$$

Here, *F*_*i*_ describes the second set of components in our CPG network, the *Pattern Formation* network that determines the temporal fine structure (shape) of the controlled variable. *a*_*n*_ and *b*_*n*_ (*n* = 0, 1, 2, …, *L* and *b*_0_ = 0) represent the first *L* Fourier series coefficients that are calculated for the corresponding *i*th joint angle time course. The coefficients are obtained using Fast Fourier Transformation (FFT) and the fundamental frequency is given by *f*_*k*_ which is 2π∕*T*, where *T* is the time span of the data set used for computing the FFT. *M*_*i*_ is a scaling factor which normalizes the output pattern. Note that with this architecture, it is possible to generate arbitrary patterns, including simple geometric waveforms such as a triangular waveform. The proposed CPG circuitry for driving the stick insect head and antennae is shown in Figure 1A. Given the fact that the stick insects show active, rhythmic yaw movements of the head (Volker Dürr, unpublished observation), it is likely that the neck joint is controlled via a central CPG and it may be coupled to the left and right antennal CPGs. A single oscillator is used to control each joint. These oscillators are mutually connected via excitatory couplings (Equation 2) with adjustable strengths (*w*_*ij*_) and phase biases (ϕ_*ij*_), and are numbered from left to right. The connection matrix is given in Table 2. For instance, the desired phase difference and the coupling strength of the connection from the neck joint (Oscillator 3) to the right HS joint (Oscillator 4) are *w*34 and ϕ34, respectively.

**Table 2:** **Connection matrix of the oscillator network shown in Figure 1A**.

**Oscillator**	**1**	**2**	**3**	**4**	**5**
1	−	ϕ12, w12	−	−	−
2	−ϕ12, w21	−	ϕ23, w23	−	−
3	−	−ϕ23, w32	−	ϕ34, w34	−
4	−	−	−ϕ34, w43	−	ϕ45, w45
5	−	−	−	−ϕ45, w54	−

**Figure 2:**
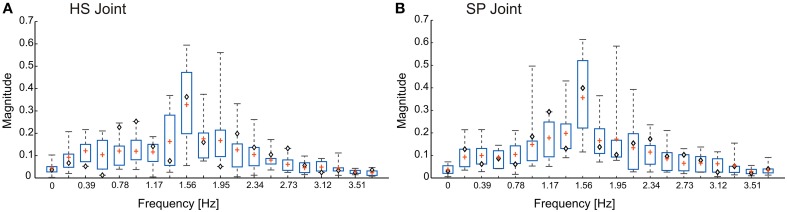
**Frequency spectra for the antennal joint angle time courses**. Both head-scape **(A)** and scape-pedicel **(B)** joints have the dominant frequency component at around 1.56 Hz. Mean values are represented by the sign +. Whiskers show the minimum to maximum range. Boxes show the inter-quartile range. The values for the model variant *M*_*c*_ are shown by ◇ signs. The total data set comprises *n* = 10 trials from five animals, with two trials per animal (data from Krause et al., 2013).

We tested the model with three different pattern formation networks, all of which were designed according to measurements on unrestrained walking stick insects. To give an impression of the variation of the real antennal movement patterns, Figure 2 shows the amplitude spectra [|*z*_*n*_|, where *z*_*n*_ = (*a*_*n*_, *b*_*n*_)] of the joint angle time courses for five different animals (two trials per animal). The frequency components of the three pattern formation networks [*F*_*i*_(θ_*i*_, *f*_*k*_)] were set according to these spectra. Since the sampling rate of the experimental data was 50 Hz, we set the time span, *T*, to 5.12 s, resulting in 256 discretized points for the computation of the FFT. Only the first 20 frequency components are shown in the figure, and the first 16 (= *L*) were chosen for the pattern formation network. The root mean squared error (RMSE), between the original data and the time course produced with the first 16 frequency components, was less than 3°. Since this RMSE is less than the natural amplitude variation, we concluded that 16 components were sufficient to faithfully reproduce natural time courses. The three variants correspond to (i) a single representative trial (◇), (ii) mean frequencies from all 10 trials (+), and (iii) mean frequencies with added variability of one standard deviation. From here onwards, we denote the three CPG patterning networks as *M*_*c*_, *M*_*m*_ and *M*_*msd*_, respectively. Additionally, stochasticity was introduced to the amplitude variable (μ_*i*_) of all three models. Statistical data for the variation of upper and lower bounds of the joint angle time courses were taken from Krause et al. (2013).

The parameters used in the rhythm-generating (oscillator) network of the CPG model are tabulated in Table 3. “U” and “L” represent the standard deviations of the variation in upper and lower bounds of the corresponding amplitude variable, respectively. The frequency and amplitude parameter values are the same for both antennal joints, but the offset values are given only for the left antenna (sign inversion for the right antenna). A negative offset of the HS joint corresponds to a dorso-lateral movement of the scape, whereas a positive offset of the SP joint corresponds to a ventro-lateral movement of the compound segment pedicel+flagellum. Due to small amplitudes in head movements, a sinusoidal pattern was used for the neck oscillator. All the network connection parameters were held fixed within a single simulation trial (ϕ_12_ = −π∕9, *w*_12_ = *w*_21_ = 20, ϕ_23_ = −π∕3, *w*_23_ = *w*_32_ = 1, ϕ_34_ = 4π∕3, *w*_34_ = *w*_43_ = 1, ϕ_45_ = π∕9, *w*_45_ = *w*_54_ = 20). Here, the phase biases were chosen to match the average values from real experimental data (Krause et al., 2013). Note that the phases of left and right HS joints (across the neck joint) are 180° apart. A suitable set of weights was obtained by systematically increasing the ratios *w*_12_:*w*_23_ and *w*_45_:*w*_34_ (both are equivalent, due to left-right symmetry) so as to reduce the circular variance of the already set phase biases ϕ_12_ and ϕ_45_. Trials with ratios of above 15 had a circular variance of less than 0.15 and showed no noticeable distortion of the antennal tip trajectory. Throughout this study, the main focus was laid on the connectivity between SP and HS joint oscillators (intra-limb coupling).

**Table 3:** **Parameters of the CPG network**.

**Oscillator**	**Head-scape**	**Scape-pedicel**	**Neck**
Frequency, *v*_*i*_ (Hz)	1.45 ± 0.05	1.45 ± 0.05	1.25 ± 0.02
Amplitude, μ_*i*_ (°)			
- Intact	39, U(08) L(09)	31, U(09) L(15)	6, U(1) L(1)
- Ablated	55, U(16) L(12)	50, U(13) L(18)	6, U(1) L(1)
Offset, *C*_*i*_ (°)			
- Intact	−60	22	0
- Ablated	−72	15	0
$\gamma_{i}=\gamma_{i}^{c}$	20	20	50

### 2.4. Computer simulation

The simulator was programmed using Python scripts and a set of open source libraries for Python. The stick insect head-antennal system was simulated using the open-dynamics engine (ODE, http://www.ode.org), which is an open-source, high-performance library for simulating rigid body dynamics. 3D graphical animation was done by means of a separate program module using OpenGL (http://pyopengl.sourceforge.net) and the interface was written using Qt libraries (http://www.riverbankcomputing.co.uk/software/pyqt). The system of differential Equations (1–3), which characterizes the oscillator network, was implemented using the second order *Runge-Kutta* method with a 5 ms time-step. The angular position control of a joint (as in a servo motor) was achieved by setting the velocity of the motor proportional to the difference between the CPG output (target angle) and the current angle value. The proportional constant and the maximum torque (a motor parameter in ODE) were set such that the RMSE between the target and the reached values was minimized: In our simulation, RMSEs for HS and SP joint angle time courses were about 1.5 and 0.6°, respectively, which is negligible compared to their respective working ranges. This allowed the dynamic model to accurately replicate behaviorally measured kinematic data. A modular structure and the object-oriented programming were used to increase the changeability and readability of the code. In particular, the controller module (the CPG and other necessary classes and functions for regulating the joint motors) that provides the reference values for the joints can be replaced by a more detailed artificial neuronal network, or by a simpler and more abstract set of sinusoidal oscillators. Moreover, a muscle model or sensory feedback can be incorporated easily into the skeletal model for further experiments.

## 3. Results

**Figure 3:**
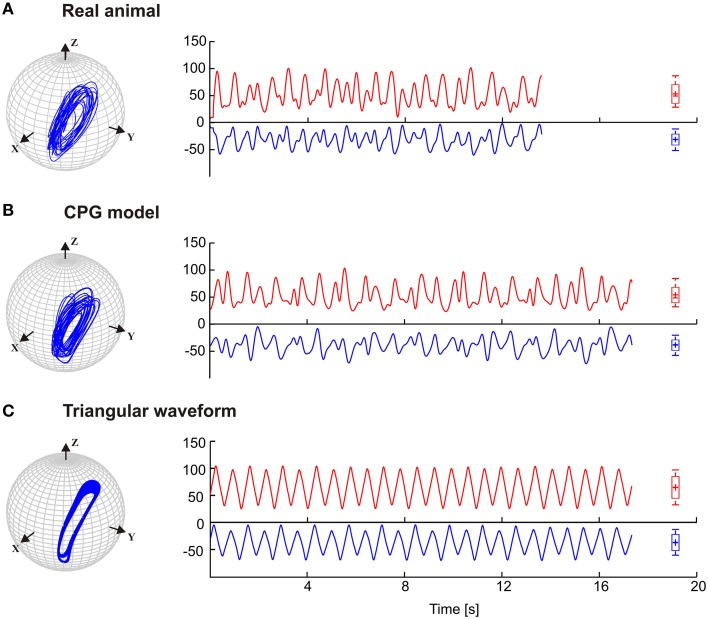
**Antennal tip trajectories and joint angle time courses of an intact animal**. HS and SP joint angles are shown by red and blue curves, respectively. **(A)** Data of a real animal as measured by Krause et al. (2013). In **(B)**, the CPG includes the pattern generators modeled according to frequency components of the real data. **(C)** Simulations with the dynamic model being driven by a simple triangular waveform for each joint. Box-Whisker plots show joint angle distribution and the 5-to-95 percentiles of the working range. For this and the following figures, angle values are given in degrees.

Stick insects show persistent elliptical antennal movement trajectories during walking. Here, we developed a 3D dynamic model of the active tactile system of the insect for the investigation of antennal motor control. In a first set of experiments, we tested the suitability of a purely CPG-driven model for simulating the natural antennal movement pattern during walking. For this, Figure 3 shows a comparison of the antennal tip trajectories and corresponding joint angle time courses of a representative experimental trial (Figure 3A) and of the model, using two driving signals of the pattern formation network. In Figure 3B, the driving signal is composed according to the frequency characteristics of the experimental joint angle time courses (single trial model, *M*_*c*_). In Figure 3C, a triangular waveform was used. The latter was chosen because it could be generated by the simplest Fourier series that still captured the fast changes in movement direction observed in the experimental data. Even though the natural antennal movement sequence clearly has rhythmic components, its rhythmicity is varying with respect to amplitude and frequency even within individual trials. Thus, it can be considered as a quasi-rhythmic movement. This quasi-rhythmicity cannot be simulated with a constant triangular driving signal. On the other hand, the similarity of Figures 3A,B show that our model can indeed simulate experimental data quite well if the pattern formation network generates a sufficiently complex driving signal to the rhythm generating network. Note that due to the similarity in searching pattern in both antennae, only cases for the left antenna are shown in Figure 3 and the following figures.

**Figure 4:**
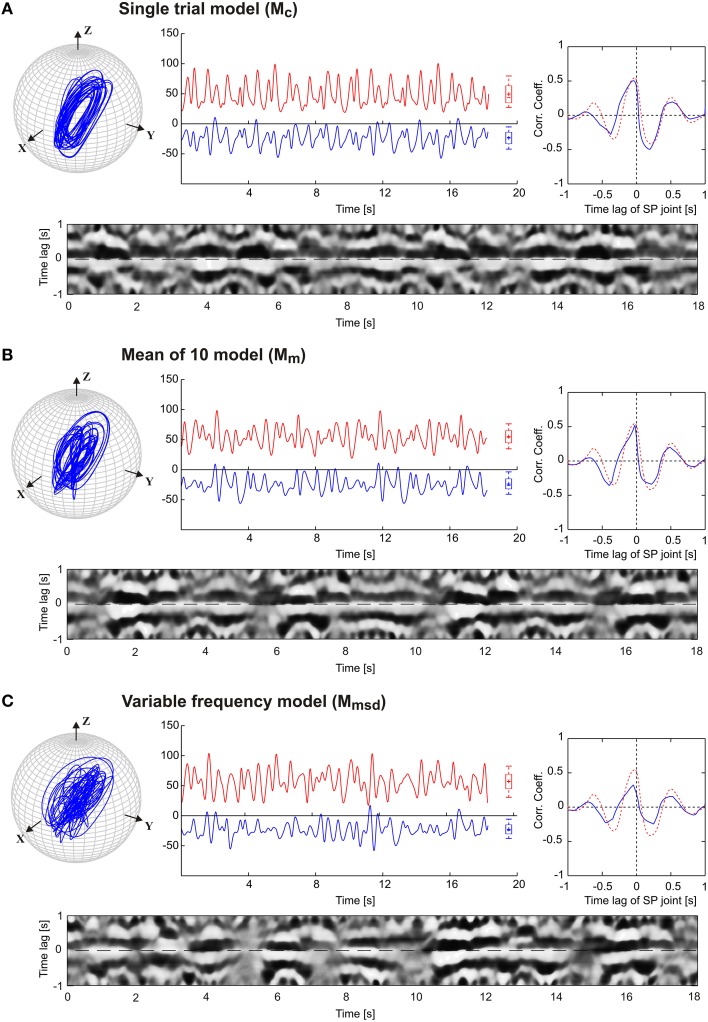
**Comparison of three pattern generator variants**. The four graphs per panel **(A,B,C)** show the tip trajectory (top left), joint angle time courses (top middle; HS: red, SP: blue), the mean cross-correlogram (top right), and the sliding cross-correlogram (bottom). The latter plots the cross-correlation of the SP joint angle relative to the HS joint angle within a sliding window size of ±1 s. Each column of the image shows the cross-correlogram for a single time window (aligned on the center of the window), with the correlation coefficient coded in grayscale from white (*r* = 1) to black (*r* = −1). The mean cross-correlograms show the average correlation coefficient for each row of the sliding cross-correlogram below. For comparison, the dotted red curve shows the average values of mean cross-correlograms for the experimental (intact animals) data (*n* = 10).

The results in Figure 3 are a proof of principle, showing that a set of coupled CPGs can produce realistic, rhythmic but quasi-rhythmic antennal movements in the skeletal model. Next, we were interested to test whether the model could capture two important characteristics seen in the experimental data: First, experimental data reveal a stable phase difference between the SP and HS joint angle time courses that varies only a little (approximately 20°), with the SP joint leading the HS joint. Second, the experimental data show considerable trial-to-trial variability of the movement pattern, both within and among individual animals (see variability of frequency spectra in Figure 2). Thus, in a second set of experiments, three CPG model variants were compared and they differed with respect to the incorporation of frequency spectra of different trials. In the single-trial model, *M*_*c*_, only the frequency spectrum of a single trial was incorporated into the pattern formation network. The mean frequency (mean of 10) model, *M*_*m*_, considered the mean frequency spectrum of 10 trials from five animals. Finally, the variable frequency model, *M*_*msd*_, incorporated the mean frequency spectrum with added random variation in the range of the standard deviation observed in the experimental data. In order to test for the phase difference between the antennal joints, the same data analysis was used as done by Krause et al. (2013). Accordingly, each panel of Figure 4 shows the antennal tip trajectory, joint angle time courses, cross-correlograms, and mean cross-correlograms for a given model variant. For immediate comparison with the experimental data, the mean cross-correlogram of the experimental data is shown as well (red dotted curves in Figure 4). A characteristic of antennal movements in intact animals is the elliptical shape of the antennal tip trajectories. While both the *M*_*c*_ and *M*_*m*_ models could generate the characteristic shape of the trajectory, the pattern obtained from the *M*_*msd*_ model strongly differed from the experimental data. The mean of 10 model (*M*_*m*_) produced antennal tip trajectories in the form of filled ellipsoids (Figure 4B). In all three model variants, the phase lead of the SP joint ϕ_*ij*_ was set to a constant of 20°, as can be seen in the experimental (real) data. Note, however, that the true, instantaneous phase difference depends on the corresponding coupling strength *w*_*ij*_, and the spontaneous amplitude of the joint angle oscillations, which may vary from one period to another. Cross-correlation of the two joint angle time courses uncovered the pre-set inter-joint coupling in model variants *M*_*c*_ and *M*_*m*_. As in the real stick insect, the SP joint was leading the HS joint, as can be seen by a negative time lag of the SP joint angle relative to the HS joint (in Figure 4, note the continuous or almost continuous horizontal white band at negative time lag in the sliding cross-correlograms). This persistent pattern of inter-joint coordination was lost in the case of variable frequency model *M*_*msd*_ (see Figure 4C). Accordingly, the mean cross-correlogram for this model variant had much lower correlation coefficients than that of the experimental data. In comparison, the two other model variants had very similar mean cross-correlograms and their match with the experimental values was much better.

**Figure 5:**
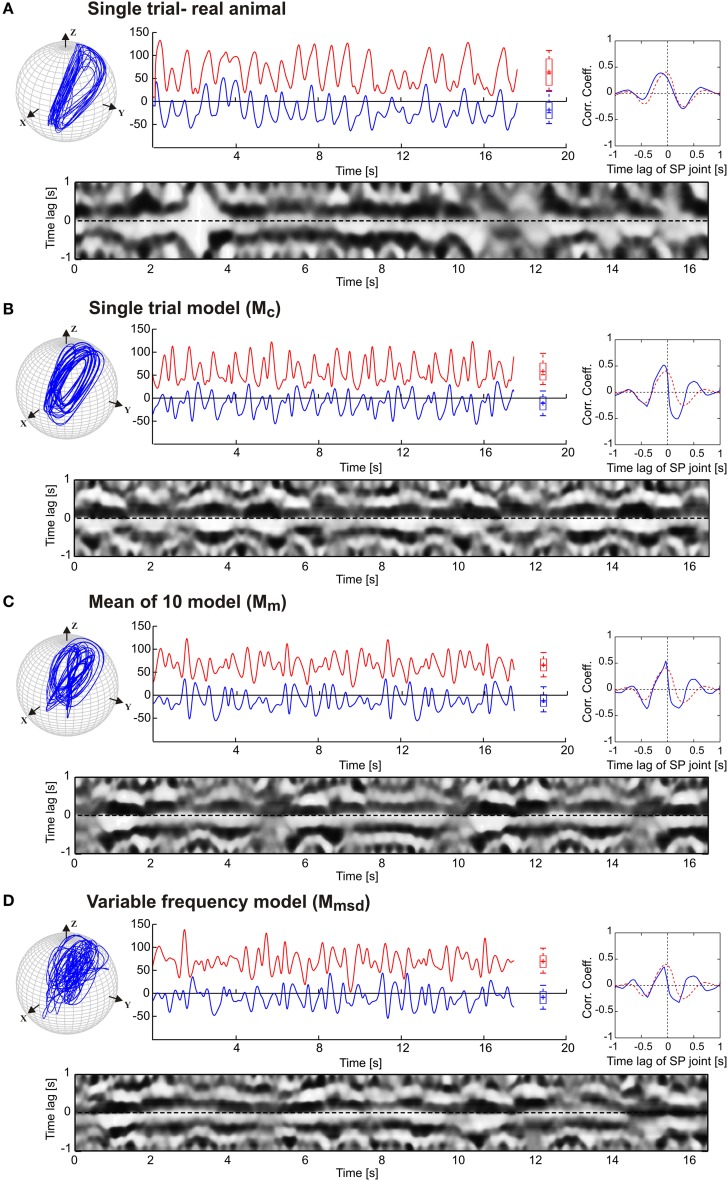
**Simulation of hair field ablation experiments**. **(A)** Representative trials of the original data from Krause et al. (2013). **(B,C,D)** follow the same arrangement and graphics details as described for Figure 4. In comparison with the simulation of intact antennae (Figure 4) only the amplitude μ_*i*_ and offset *C*_*i*_ (mean angle of oscillation) of the oscillators were changed according to the average values obtained from experimental data. The parameter values are tabulated in Table 3. The dotted red curves in the inserts show the average cross-correlogram for the data from *ablation* experiments (*n* = 5).

Next, we tested to what extent our model was able to simulate experimental data on animals with sensory ablations. For this, we used data on antennal movement after sequential ablation of all dorsal antennal hair fields, as reported by Krause et al. (2013). Figure 5 shows the simulated antennal tip trajectories and the joint angle time courses for each model, along with the same correlation analysis as used in Figure 4. The main effect of the ablation of these antennal proprioceptors was a strong increase in the working ranges of both antennal joints of the animals, as illustrated by the much wider ellipses of the antennal tip trajectories in Figure 5 (top). Hair field ablation experiments were simulated using the same three model variants as for intact animals, i.e., the pattern formation networks were exactly the same as before. The only difference was that the simulation of the ablation experiments required a change of the amplitude and offset settings of the oscillators controlling each joint (see Table 3). In all three models, the increase in amplitude of both joint oscillators resulted in the broadening of the antennal searching space. Despite the similarity in working ranges, the trajectory of the variable frequency model *M*_*msd*_ again differed from that of the other two models; *M*_*c*_ and *M*_*m*_ models showed relatively persistent patterns in the tip trajectories, whereas *M*_*msd*_ did not. Similar to the simulation of intact antennae, the cross-correlograms revealed a persistent phase coupling between SP and HS joints in *M*_*c*_ and *M*_*m*_ models. With regard to the mean cross-correlograms, the difference between the model and the experimental data was slightly larger for ablation experiments than in the intact situation (Figure 4). There were two reasons for this: first, the frequency spectrum used for the model was set according to data on intact animals; second, the phase difference varied slightly more in animals with hair field ablations compared to intact animals. A relatively low and broader central peak of the mean cross-correlogram (red dotted curve in Figure 5) of the ablation experiments stems from an overall shift of the frequency spectrum to lower frequencies, together with additional variability. Note that up- or down-scaling the frequency spectra of the *M*_*c*_ model by 50% did not change the characteristic shape of the antennal tip trajectory (see Supplementary Figure 1). Therefore, we used the frequency spectrum of intact animals for simulating the ablation experiments as well.

**Figure 6:**
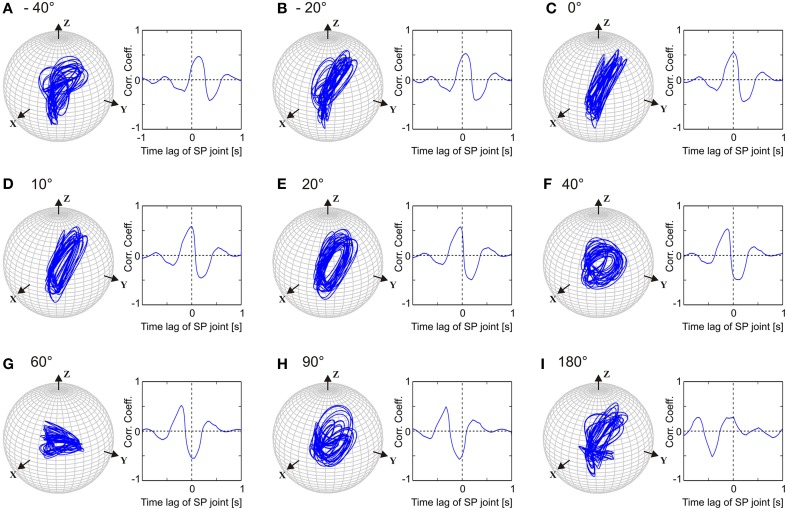
**Comparison of left antennal tip trajectories obtained after varying the phase difference between HS and SP oscillators in *M*_*c*_ model**. In each sub-figure, tip trajectory (left) and the mean cross-correlogram (right) are shown for a single simulated trial. See Figure 5 and text for more details. Phase lead of the SP joint with respect to the HS joint varied from −40 to 180°, as shown in **(A–I)**. The natural situation is where the SP joint leads HS joint by 20° **(E)**.

Like walking stick insects, our model showed persistent inter-joint coordination in antennal movement, with the SP joint leading HS joint by approximately 20°. Thus, we were interested in understanding how important this phase difference is for generating the typical antennal movement pattern. Owing to the complex shape of the patterns, their quasi-rhythmicity (variability), and the non-orthogonal slanted axes of antennal joints, it is not trivial to predict how the magnitude of the model parameter setting, the phase difference, i.e., ϕ_*ij*_ in Equation (2), will affect the antennal tip trajectory or the joint angle time courses. In order to investigate the significance of this parameter, we systematically varied ϕ_*ij*_ in the single-trial model variant *M*_*c*_. Figure 6 shows separate simulations with the phase lead of the SP oscillator ranging from −40, −20, 0, 10, 20, 40, 60, 90, and 180°. Judging from the antennal tip trajectories and mean cross-correlograms for HS and SP joint angle time courses, the antennal movement pattern increasingly deviated from the natural reference condition ($\phi_{ij}=20^{{}^{\circ}}$) as the phase lead increased or decreased. If the phase difference had been irrelevant to the movement pattern, we would have expected an elliptical tip trajectory for all values of ϕ_*ij*_, with varying width and orientation. Instead, the elliptical shape of the movement pattern could be seen only within the limits of approximately 10° above or below the natural reference (i.e., from 10 to 30° lead). Moreover, when the two joint oscillators were in phase ($\phi_{ij}=0^{{}^{\circ}}$), we would have expected the antennal tip to move along a slanted line. Instead, due to the variability introduced into the pattern generators, antennal tip trajectories ran along several lines (Figure 6C). In order to determine whether this is true for single-trial models simulating the data from other individuals, we repeated the same experiment with different Pattern Formation networks (Equation 5), i.e., after setting the frequency characteristics (Figure 2) according to the frequency spectra measured from four further animals. In all of these cases, we found the same result: the antennal movement pattern substantially deteriorated for phase differences beyond ±15° with regard to the natural reference.

**Figure 7:**
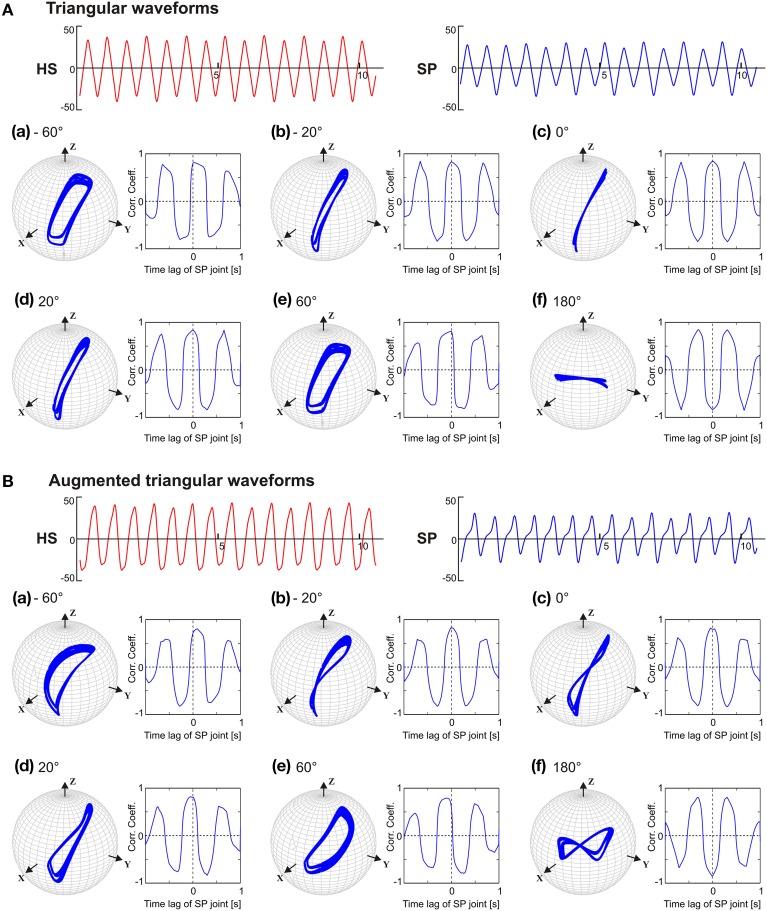
**Phase sensitivity of antennal tip trajectories with the pattern-generating network producing the same frequency spectrum but distinct phase spectrum**. In **(A)**, the oscillators were driven with a symmetric triangular waveform (sine function only). In **(B)**, the driving waveform contained the same frequency components but differed in phase (sines and cosines). As in Figure 6, the phase lead of the SP joint with respect to the HS joint varied from −60 **(a)** to 180° **(f)**. Units for abscissa and ordinate for the HS and SP waveforms are seconds and degrees.

Having observed that the phase lead between the SP and the HS joint is a limiting factor for obtaining the typical, elliptical shape of the antennal tip trajectory, we wondered which aspect of the pattern-generating network could be the reason for this limitation. Therefore, we tested the CPG model with two simple patterns of oscillation that contained the same four frequency components but differed in phase. When the joints were driven with symmetrical triangular waveforms with unique phase (e.g., sine functions only), the antennal tip trajectories were elliptical with flat ends along the major axis, except if the phase differences was 0 or 180° (see Figure 7A). In the latter two cases, i.e., in-phase (**c**) and anti-phase (**f**) coordination, the antennal tip moved along a slanted vertical or horizontal line, respectively. When the phase difference between the joint oscillators increased, the width of the elliptical trajectory increased, irrespective of which joint was leading or lagging. A similar effect could be seen with frequency-scaled triangular waveforms (see Supplementary Material). Antennal trajectories showed a more complex dependency on phase, as soon as driving waveform contained two distinct phases per joint. Figure 7B shows the effect of augmented triangular waveforms, where the same four frequency components were present as both sine and cosine terms (phase shifted) in its Fourier representation (Equation 5). As a consequence, HS and SP joint angle time courses had slightly asymmetric positive and negative halves (see Figure 7B). With regard to the phase dependency of the antennal tip trajectory, the main effect induced by the augmented waveform was a distinct change in trajectory shape from an ellipse to a figure-eight shape (Figure 7B). In summary, as soon as the pattern-generating network produced waveforms with more than one phase in its phase spectrum, even simple composite waveforms resulted in distinct antennal tip trajectories as soon as the phase difference between the two oscillators changed.

## 4. Discussion

For adaptive autonomous behavior, the active tactile system plays a major role in insects, because it is a dedicated sensory structure to explore and interact with the near-range environment. Generally, active tactile exploration behavior is divided into searching (e.g., mammals: Towal and Hartmann, 2008; Arkley et al., 2014; insects: Okada and Toh, 2004; Krause et al., 2013) and sampling (e.g., mammals: Ahissar and Knutsen, 2008; Zuo et al., 2011; insects: Harley et al., 2009; Schütz and Dürr, 2011; Krause and Dürr, 2012), depending on whether or not the sensor(s) is (are) making contact with an object. Here, we focus on the searching aspect of active tactile exploration, although the model is sufficiently general for being applied to tactile sampling in subsequent studies.

The model that we propose consists of a set of mutually coupled CPGs that, at the network level, are responsible for generating the rhythmic antennal movements of the stick insect. We chose the computational framework to lie between two opposite extremes: (i) On the one side, time-driven trigonometric waveforms or phase-coupled oscillator models have been used to further our general understanding of coupled oscillator systems. However, these systems neglect the neuronal dynamics and have little or no capability of making predictions associated with neural rhythm generation (Kopell and Ermentrout, 1988; Ijspeert, 2001). (ii) At the other extreme, detailed biophysical models have been developed that incorporate detailed descriptions of known neurons and pathways (Kozlov et al., 2009; Daun-Gruhn, 2011). These models require a substantial knowledge about the underlying network physiology and still can face the problem of losing the influence of individual parameters within the complexity of the models. In a previous study, an Echo State Network (ESN) was used directly control movements of the antenna in a simulated hexapod (Krause et al., 2009). The model was able to produce different rhythmic trajectories, including a smooth transition between two distinct movement patterns. Even if an ESN model could be trained to produce quasi-rhythmic movements, there is no clear division into distinct oscillator networks for distinct joints, neither are any movement parameters explicitly coded into the network. Since the present study aimed at a systematic understanding of the key parameters of coupled quasi-rhythmic movement generators, both of the latter properties were required. With an ESN approach, the weights of the network have to be trained so that the network output can replicate the experimental dataset. As a result, one has little or no control over particular oscillator parameters before the resulting output is known. In the present study, our primary aim was to explore and understand the effect of variation of CPG parameters such as amplitude and phase difference on the kinematics of active tactile exploration behavior, and to provide a quantitative comparison with real experimental data on tactile searching behavior (Krause et al., 2013). The use of a Hopf oscillator as the basic rhythm generator was advantageous since it has a stable limit cycle and allows independent control over the amplitude, frequency, and phase parameters. The simplifications made, including the use of servo-motor like joints and a network of simple non-linear oscillators, make this study immune to all the uncertainties involved with trying to faithfully model limb masses, joint friction, muscles and neural mechanisms underlying muscle activation patterns. As yet, the model was able to successfully simulate experimental findings on intact and proprioceptor-ablated stick insects.

In order to obtain a steady phase difference between HS and SP joint oscillations, both a *strong* connectivity between the oscillators controlling the corresponding joints (*w*_12_ = *w*_21_ = 20 in Figure 1A) and a *weaker* connectivity between the HS and Neck oscillators (*w*_23_ = *w*_32_ = 1) were advantageous but not compulsory. In fact, this weaker coupling via the neck oscillator will allow to generate largely independent movements by the two antennae. The inclusion of the neck oscillator was inspired by the fact that the stick insects show persistent rhythmic yaw movements of their head while walking (Volker Dürr, unpublished observation), much like walking flies do (Kress and Egelhaaf, 2012). Therefore, connectivity between the neck oscillator and the oscillator networks driving each antenna is vitally important for obtaining coordinated head movements. Although the present study focused on the inter-joint coordination within a single antenna only, the coupling between the head (i.e., the neck joint) and the antennal movements has already been included into the controller framework and the skeletal model (see Supplementary Material, Section 1.2). This will allow the expansion of the active tactile exploration system by active head movements and, thus, provide insight into the role of the head in increasing the volume searched by the antenna.

### 4.1. Effect of proprioceptor ablation

There are several mechanosensory sub-modalities that contribute to the tactile perception in insects (hair fields, tactile hairs, companiform sensilla, and Johnston's organ, Dürr, 2014). The hair fields are considered as proprioceptors as they encode the (actively controlled) posture of a limb (Wendler, 1964) and are common joint proprioceptors in insects, as reviewed in Staudacher et al. (2005). In stick insects, ablation of all dorsal hair fields on the antenna mainly affects the working ranges of both antennal joints, with little to no effect on the phase difference between the joints (see below). As can be seen in Figure 5, the effect of the hair field ablations can be simulated by changing the amplitude (μ_*i*_) and the offset (*C*_*i*_) of the corresponding joint oscillators, only (see Supplementary Movie 1). Accordingly, the observed increase in working range can be described as a result of either impaired position control or a reduction in effective joint stiffness due to the alteration in local proprioception. Indeed, hair fields have been shown to be involved in negative feedback loops controlling the position of antenna, for instance in flying locusts (Saager and Gewecke, 1989; Bauer and Gewecke, 1991) and moths (Krishnan et al., 2012). The same function has been shown for a hair field at the Coxa-Trochanter joint of the stick insect legs (Schmitz, 1986; Büschges and Schmitz, 1991). For a leg joint, Zakotnik et al. (2006) showed that load compensation can be enhanced by regulating the joint stiffness via co-contraction of antagonist muscles, together with high levels of passive forces. Taking into consideration this evidence, we propose that the antennal hair fields control the local effective stiffness of each joint by acting through negative feedback loops. Possible pathways could either act directly on the motoneurons of the muscles actuating the corresponding joint, or act on interneurons that may be part of the CPG network driving these muscles.

### 4.2. Model variants

As for the joint kinematics of the individual animals, a substantial variability could be seen in the frequency spectra of the antennal joint oscillations (see Figure 2). However, the dominant frequency varied only within a small range from 1.36 to 1.95 Hz. The core idea behind the experiments with two model variants that include experimental variability (Mean of 10 model, *M*_*m*_ and Variable frequency model, *M*_*msd*_) was to develop a generalized CPG model accounting for variation in frequency characteristics of the joint angle time courses among different individuals. Antennal tip trajectories generated by the single trial model (*M*_*c*_) and the *M*_*m*_ variant were similar in simulations of both intact animals (Figure 4) and those with hair field ablations (Figure 5). In contrast to these two model variants, the *M*_*msd*_ model failed to simulate antennal joint kinematics in both cases. One possible reason could be that the variability introduced to the frequency components in the pattern formation network (*F*_*i*_ in Equation 5) was higher than the normal variation in the frequency spectra for joint angles in an individual animal. In the model, variance was computed after pooling the angle data from all the animals. It is possible that the shape of the frequency spectrum is characteristic for a given individual, while it could differ significantly from one animal to another.

### 4.3. Stable phase difference despite quasi-rhythmicity

From Figure 6, it is evident that the phase difference between the SP and HS joint angle time courses plays an important role in generating the elliptical movement pattern of the antennal tip. Given the fact that an elliptical trajectory improves tactile efficiency (Krause and Dürr, 2004), this suggests that an appropriate phase difference between the antennal joints is of functional relevance to tactile exploration behavior. According to our simulations, the *phase lead* of the SP joint could vary from about 10 to 30° without disrupting the elliptical pattern. Interestingly, if the phase coupling was reversed, i.e., the SP *lagged* the HS joint by 20°, the model could no longer produce the experimentally observed pattern (for an explanation, see below; Supplementary Movie 2 illustrates the effect). Experiments on stick insects have shown that the movement of the two joints remains strongly coupled even after ablations of proprioceptive hair fields (Krause et al., 2013), and that the pattern of inter-joint coordination remained the same: the SP joint always lead the HS joint. Furthermore, these experimental results showed that the effect of hair field ablations on the magnitude of the phase difference was always small, albeit statistical significance in some conditions. In all conditions tested experimentally, the reported changes were within the range for which our simulation results show little or no effect on the antennal tip trajectory. This supports the view that in stick insects, the phase difference necessary for maintaining an elliptical antennal tip trajectory despite quasi-rhythmicity is coded into the pattern-generating network, and is affected only little by proprioceptive hair fields.

We demonstrate that the disruptive effect of increasing phase difference on an elliptical trajectory is not due to the presence of multiple frequency components but is consequence of the phase spectrum. When the joint oscillators were driven with a triangular waveform equivalent to the sum of weighted first harmonics (mostly odd) of a sinusoid, the elliptical pattern of the antennal tip trajectory remained unaffected by the phase difference between the joints (Figure 7A). Since this result was obtained with the same skeletal model, including non-orthogonal joint axes (see Section 2.2) and the separation of the joint axes by the length of the scape, these morphological properties cannot be the decisive factor. However, experiments with the augmented triangular waveforms in which weighted harmonics of both cosines and sines had been included, revealed a strong effect of increasing phase difference between the joints on the antennal tip trajectory (see Figure 7B). The addition of cosines in the augmented triangular waveform introduced a second phase to the phase spectrum of the waveform, leading to a slight asymmetry between the positive and negative halves. On the other hand, scaling up (or down) of the frequency spectrum of the triangular waveforms while keeping the original phases intact, did not show the phase sensitivity of the antennal tip trajectory (see Supplementary Figure 2). Furthermore, scaling (50% up and down) of the frequency spectra for both HS and SP joint oscillators (in *M*_*c*_ model) did not affect the shape of the antennal tip trajectory pattern (see Supplementary Figure 1). In addition, when the antennal joints were driven by a modified *M*_*c*_ model with *zero or unique* phase spectrum, while leaving the frequency (amplitude) spectrum unchanged, an increase in the phase difference between the joints did not disrupt the antennal tip trajectory (see Supplementary Material, Section 1.3). Thus, we conclude that the limiting factor for the phase difference between HS and SP is the complex shape and the dissimilarity of the joint angle time courses, reflected in different phase spectra.

In neurophysiological studies, there are many examples where neural circuits are involved in maintaining constant phase differences among neural oscillators in order to generate coordinated movements. One such neural network is the locomotor CPG involved in generating swimming behavior in lower vertebrates and invertebrates. The spinal cord of lamprey, salamander, and *Xenopus* consist of many, serially homologous segments or network elements that produce a quasi-sinusoidal wave that travels along the body with a constant phase difference while maintaining one wave-length from front to rear. This has been addressed both experimentally (Kotaleski et al., 1999; Grillner, 2006) and computationally (Kopell and Ermentrout, 1988; Cohen et al., 1992; Bicanski et al., 2013). Based on experimental data, models have been developed for swimming in leech (Cang and Friesen, 2002) and for crawling in *Drosophila* larva (Gjorgjieva et al., 2013). In both cases, the models could generate constant phase lags as observed during swimming or crawling, suggesting that some of the network motifs responsible for the generation of coordinated movements might be shared among species. Following the same reasoning, we suggest that the constant phase difference seen in the antennal joint movements is coded into the CPG. Thus, we propose that the rhythmic antennal searching-movement of the stick insect is more centrally controlled and the proprioceptors are acting locally to regulate the joint movements.

Here, we mainly focused on realistic movement generation for active tactile exploration in an insect, using a central drive only. Sensory feedback is included only implicitly by simulating the effect of proprioceptor ablations with a change in offset and amplitude. Future studies will need to include context-dependent changes in antennal movement pattern, for example during object sampling (Krause and Dürr, 2012) or during turning (Dürr and Ebeling, 2005), both of which will require two kinds of sensory feedback: proprioceptors, such as hair fields, influencing the joint angle working ranges, and exteroceptors, such as touch/contact sensors, inducing object sampling (Krause and Dürr, 2012), negotiation or avoidance (Harley et al., 2009; Baba et al., 2010). A computational model of antennal hair field function has recently been proposed (Ache and Dürr, 2015), and can be easily incorporated into the present model, e.g., by turning the offset and amplitude parameters into functions of the corresponding hair field output. Similarly, a recent model of insect-inspired tactile contour-tracing uses touch events to induce discrete shifts of the oscillator phase (Krause et al., 2014). To date, a computational model of antennal tactile hairs is not available. The current skeletal model can be extended by muscle models for compliant control, thus turning the current position control of antennal joint angles into a more realistic control by means of muscle forces or joint torques. Given the lack of neurophysiological data on insect antennal muscles, one would need to use either a simple spring-and-damper model, or adapt an existing model for insect leg muscles (Zakotnik et al., 2006; Kukillaya and Holmes, 2009; Blümel et al., 2012; Toth et al., 2013). With a muscle model, feedback from hair fields can be used to regulate the effective joint stiffness as we suggest in Section 4.1. Finally, fully functional neuro-musculo-skeletal-control system for insect head-antennal system will be useful in future experiments *in silico*.

### 4.4. Conclusions

In summary, we have proposed a modeling framework which captures the joint kinematics essential for generating rhythmic and coordinated antennal movements of the stick insect by a simple CPG architecture with a small number of parameters. The CPG consists of two networks, (i) a set of phase-coupled Hopf oscillators for rhythm generation and, (ii) a pattern formation network for capturing frequency characteristics of joint oscillations. Comparison with experimental data allowed us to derive three major conclusions: First, two variants of the proposed CPG model (single-trial variant *M*_*c*_ and mean of 10 variants *M*_*m*_) successfully produced biologically realistic quasi-rhythmic movements with a stable phase shift. Secondly, the effect of proprioceptive hair field ablation could be simulated with a change in amplitude and offset parameters, only. Finally, we found that the *phase-lead* between SP and HS joint angles plays an important role in producing behaviorally observed elliptical trajectory of the antennal tip, which in turn is known to improve tactile efficiency. We showed that this phase-lead has a limited range from about 10 to 30°, owing to the phase spectrum of the joint angle time courses. Our model provides a general framework for experiments on the arthropods head-antennal motor system.

## Funding

This work was supported by European Union (EU) grant EMICAB (FP7-ICT, grant no. 270182) to VD and by a stipend of the DFG-funded Cluster of Excellence Cognitive Interaction Technology “CITEC” (EXC 277) to NH.

### Conflict of interest statement

The authors declare that the research was conducted in the absence of any commercial or financial relationships that could be construed as a potential conflict of interest.

## Supplementary material

The Movie S1 shows an example simulation of the insect antennal model during intact and after hair field ablation. The second movie shows the effect of the phase difference between SP and HS joint oscillations on the antennal tip trajectory.
