# Supplementary material for: Stable phase-shift despite quasi-rhythmic movements: a CPG-driven dynamic model of active tactile exploration in an insect
Source: Front Comput Neurosci. 2015 Aug 21;9:107. doi: 10.3389/fncom.2015.00107 (PMC4543877; doi:10.3389/fncom.2015.00107)
Supplement: Supplementary file 3 [file DataSheet1.PDF]

Frontiers in Computational Neuroscience

Research Article
12 August 2015

Supplementary Material: Stable phase-shift despite quasi-rhythmic movements: a CPG-driven dynamic model of active tactile exploration in an insect

Nalin Harischandra [1,2,*], André F. Krause [2] and Volker Dürr [1,2,*]

[1] *Department of Biological Cybernetics, Faculty of Biology, Bielefeld University, Bielefeld, Germany*
[2] *Cognitive Interaction Technology Center of Excellence (CITEC), Bielefeld University, Bielefeld, Germany*

Correspondence*:
Volker Dürr
Department of Biological Cybernetics, Faculty of Biology, Bielefeld University, Universittsstr. 25, 33615 Bielefeld, Germany, volker.duerr@uni-bielefeld.de

Nalin Harischandra
Department of Biological Cybernetics, Faculty of Biology, Bielefeld University, Universittsstr. 25, 33615 Bielefeld, Germany, nalin.harischandra@uni-bielefeld.de

1 ADDITIONAL EXPERIMENTAL RESULTS

1.1 EFFECT OF FREQUENCY SCALING ON THE ANTENNAL TIP TRAJECTORY

In order to test the effect of a stronger change to the frequency spectrum than that used for the Mean-of-ten model, two additional simulations were carried out. For this, the single trial model (M_c) was used with an up- or down-scaled frequency spectrum (50% up and down, respectively). In both simulations, the phase spectrum was kept unchanged. We found that the characteristic shape of the antennal tip trajectory was not affected by the frequency scaling. **Supplementary Figure 1** shows the comparison with the simulation using the normal spectrum (scale factor = 1.0).

A similar experiment was carried out with the triangular waveforms used in **Figure 7**. Here, the frequency spectrum was scaled up by 50% without changing the unique phase. When the newly generated patterns were applied to both HS and SP joints of the skeletal model, the effect of changing the phase difference between the two joint oscillators remained the same as observed with the original frequency spectrum (**Figure 7A**). As the phase difference increased, only the width of the elliptical trajectory increased, irrespective of which joint was leading or lagging, except for 0 or 180° cases (**Supplementary Figure 2**). The same happened when the frequency spectrum was scaled-down by 50% (not shown). Therefore, we conclude that the shape of the trajectory is not affected by changes to the frequency spectrum, whereas it is affected by changes to the phase spectrum.

1.2 BILATERAL COUPLING OF LEFT AND RIGHT ANTENNAE

Since our model assumes that both antennae are coupled with each other via the neck oscillator, we compared the outcome of this coupling with that seen in an intact walking stick insect. For this, we analyzed head-antenna coordination of an unrestrained walking stick insect on a planar surface of 1.2 m diameter. To investigate the coupling of the yaw movements of the head with the searching movements

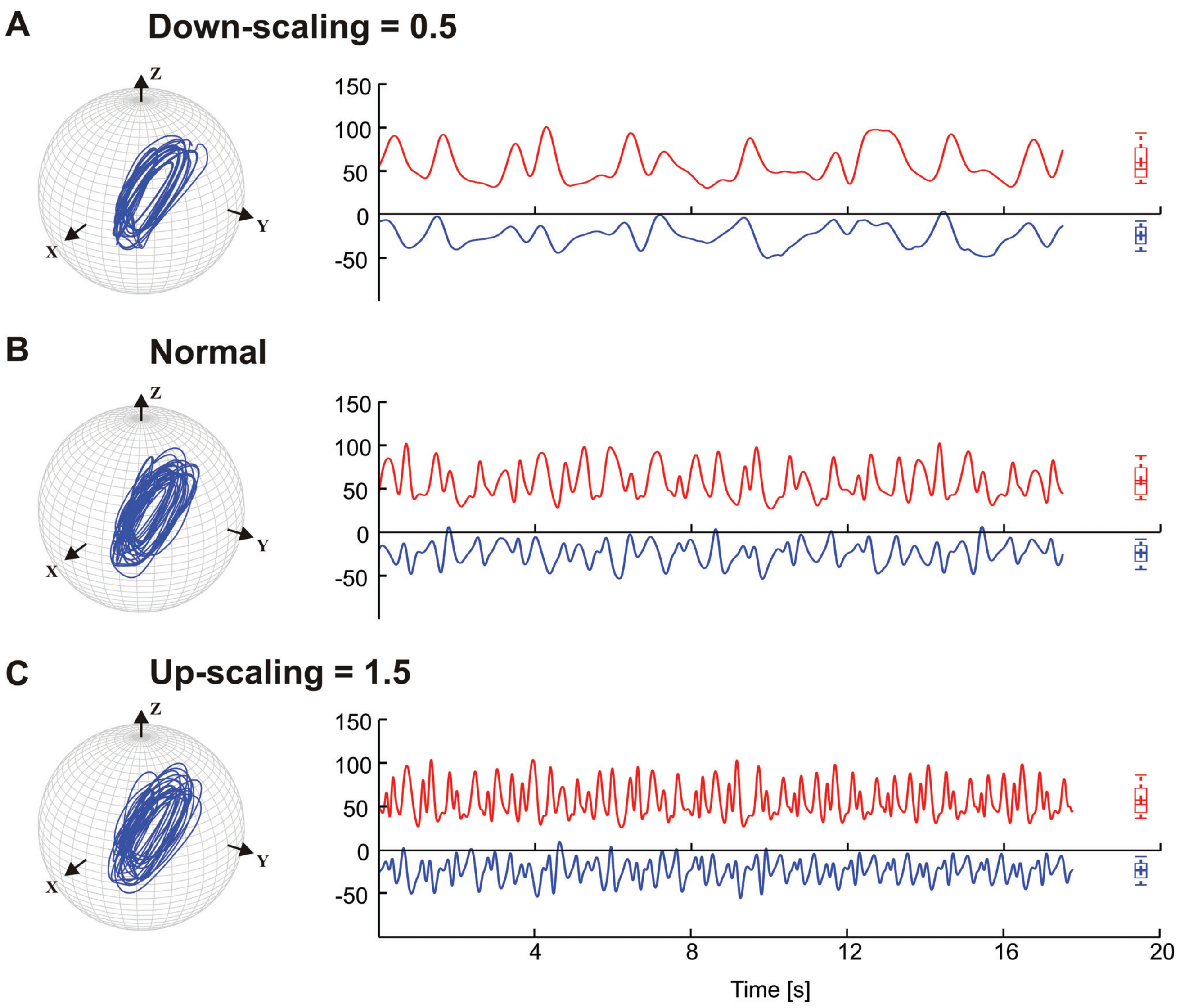

Supplementary Figure 1. Effect of scaling of the frequency spectrum in the M_c model. (**A**) and (**C**) show the left antennal tip trajectory and joint angle time courses after scaling the frequency spectrum by factors 0.5 and 1.5, respectively. The natural situation (scale factor 1) is shown in (**B**). HS and SP joint angles (in degrees) are shown by red and blue curves, respectively.

of both antennae, the projection of the flagellum onto the horizontal body plane was considered, i.e., the azimuth of antennal pointing direction. As can be seen from the time courses in **Supplementary Figure 3A**, the movements of the left and right antennae are sometimes *in phase* and sometimes *out of phase*. In other words, a drift in phase difference between the two antennae can be seen. In a cycle-by-cycle analysis with the period of the head movements serving as the reference, both the left and right antennae with respect to head yaw rotation are shown in the right panel. Note that, the peak-to-peak range of the mean antennal azimuth of either antennae was $\approx 20°$, only, which is well below the average peak-to-peak range of the antennal azimuth time course ($\approx 80°$). The much smaller range of the cycle-by-cycle average clearly underscores the observation of a continuous drift in phase coupling between head and antennae.

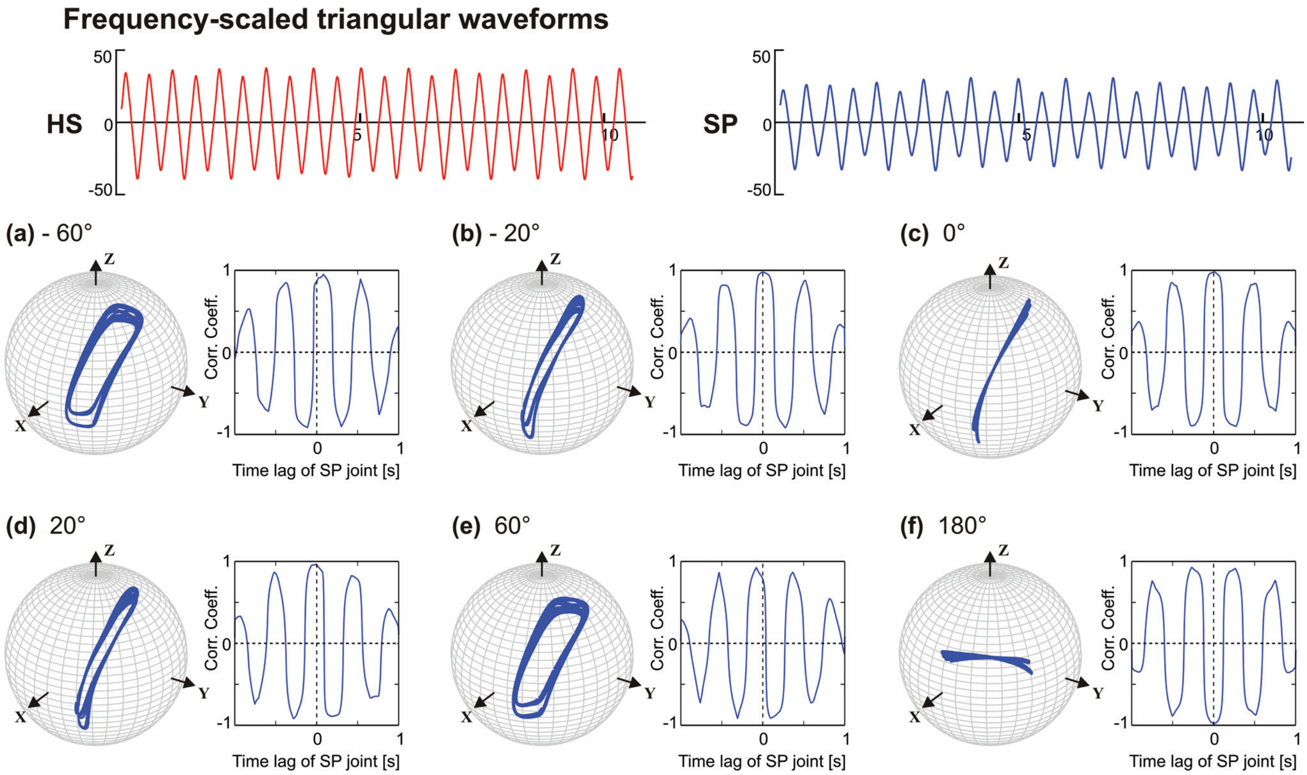

Supplementary Figure 2. Phase sensitivity of antennal tip trajectories driven by frequency-scaled triangular waveform. The oscillators were driven with the same kind of triangular waveform as used in **Figure 7A** in the main manuscript, but after scaling the frequency spectrum by 50%, while leaving the phase spectrum unchanged. The phase lead of the SP joint with respect to the HS joint varied from -60 (**a**) to $180°$(**f**). Units for abscissa and ordinate for the HS and SP waveforms are seconds and degrees.

For immediate comparison, the simulation results from the single trial model, M_c, are shown in **Supplementary Figure 3B**. The CPG model parameters were kept the same as in the simulations presented in the main manuscript, having a much weaker coupling between the neck and either HS joint than between ipsilateral HS and SP joints. The model output is very similar to the experimental data of the real animal, revealing both a drift between contralateral antennal azimuth and a much smaller range of antennal azimuth in the cycle-by-cycle average than in the natural time course. Hence, both the quasi-rhythmicity of both antennae and the drift of the phase difference between them were captured well.

1.3 PHASE SENSITIVIY OF M_C MODEL WITH UNIQUE PHASE SPECTRUM

In order to test the effect of the frequency (amplitude) spectrum on the phase sensitivity of the antennal tip trajectory, separate simulations were carried out using the single trial model M_c, with the phase spectrum set to zero, while changing the phase lead of the SP ranging from -60 to 180°. Here, only the *sine* part of the Fourier series was used (equation 5) such that all the coefficients a_n were zero and all coefficients b_n were replaced with $|z_n|$s [where $z_n = (a_n, b_n)$] so that the amplitude spectrum was not affected. The simulation results are shown in **Supplementary Figure 4**. As the phase difference increased, the width of the trajectory increased, irrespective of which joint was leading or lagging, except for 0 or 180° cases. Additionally, a 180° rotation of the trajectory can be seen when changing from lagging to leading. This is due to the change in the rotational direction. However, there was no disruptive effect on the trajectory within the phase difference range of -60 to 60°. In contrast to the results from the original M_c model (see **Figure 6(e)** and **(b)**), no distortion can be seen when the phase lead of the SP changed from 20 to -20°.

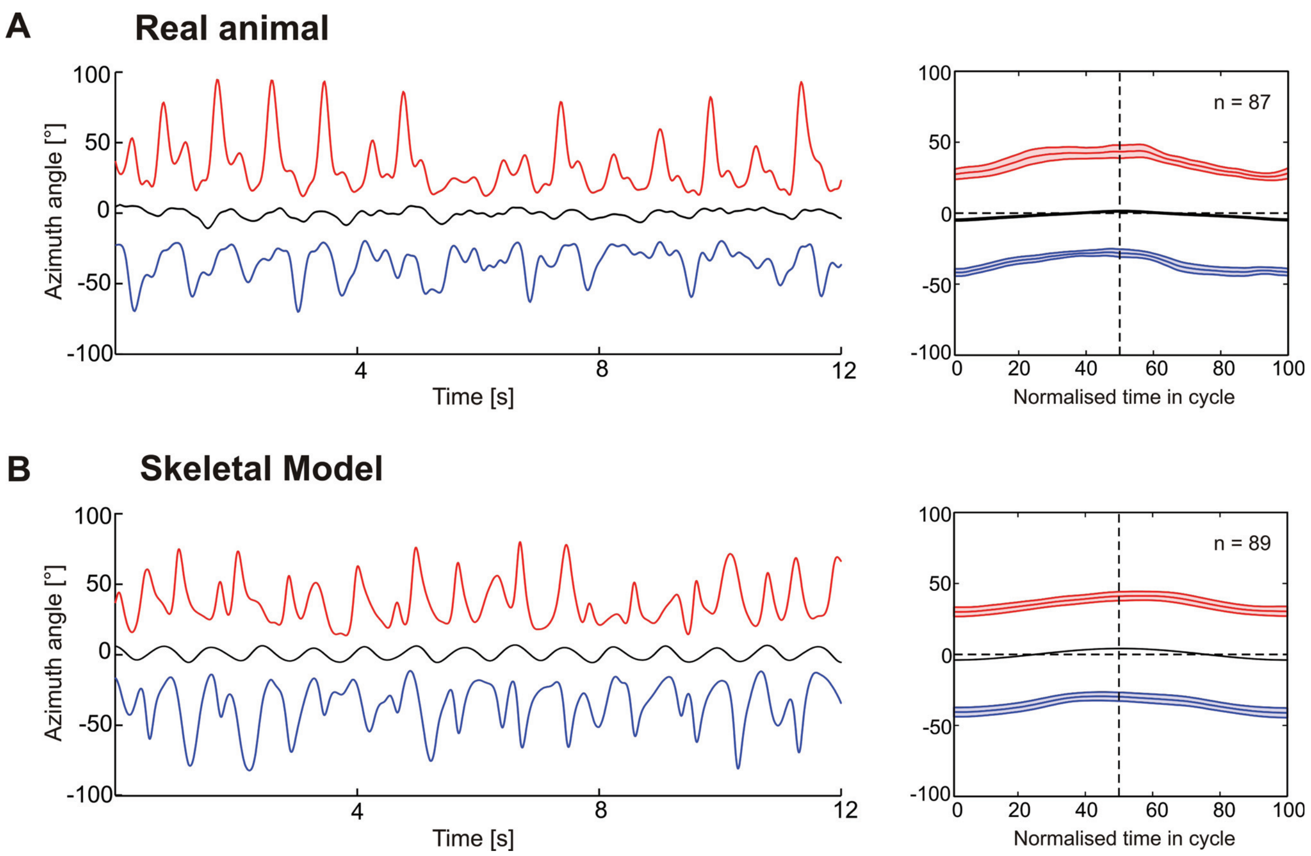

Supplementary Figure 3. Bilateral antennal movements are weakly coupled to the head. Representative time courses of antennal azimuth and head yaw rotation (left) and cycle-by-cycle analysis of head-antenna coordination (right). Experimental data from an unrestrained walking stick insect are shown in (**A**) while results from the simulation are illustrated in (**B**). In each sub-figure, the left panel shows the time courses for azimuth angles of the left (red) and right (blue) antenna, and the yaw rotation of the head (black), whereas the right panel shows the mean antennal azimuth per head movement cycle. Mean and 95% confidence intervals of the mean (shaded) are shown for all three mean time courses, using the same color code as in left panels. Owing to a drift of antennal azimuth relative to the head yaw angle, the amplitude ranges of the cycle-by-cycle means are much smaller than the real working ranges in both the experimental data and in simulation. Numbers of cycles are 87 and 89 for real and simulation data, respectively.

Taking into account the results from both models, we strongly suggest that the limitation of the phase-lead between SP and HS (to obtain the characteristic shape of the trajectory) is caused not by the frequency (amplitude) spectrum, but by the phase spectrum of the joint angle time courses. Note that the relatively low and broader peaks in mean cross-correlograms stem from the large-amplitude peaks and troughs in the time courses of HS and SP.

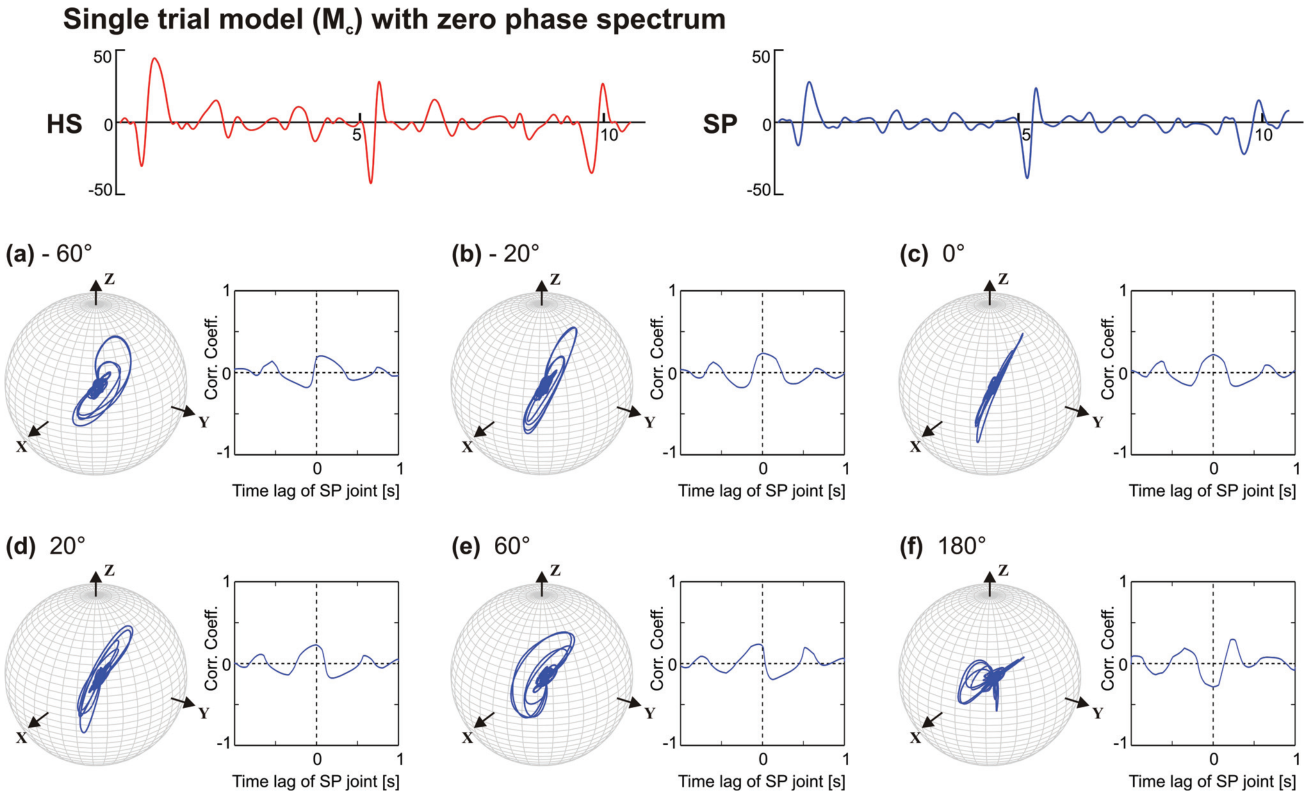

Supplementary Figure 4. Phase sensitivity of antennal tip trajectories driven by single trial model, M_c, with zero phase spectrum. The oscillators were driven with the waveforms generated by M_c model with zero phase spectrum, while leaving the frequency (amplitude) spectrum unchanged. The phase lead of the SP joint with respect to HS joint varied from -60 (**a**) to $180°$(**f**). Units for abscissa and ordinate for the HS and SP waveforms are seconds and degrees.
